# A New COL1A1 Mutation Associated With Type I Osteogenesis Imperfecta: Treatment Options for a Woman of Childbearing Age

**DOI:** 10.1210/jcemcr/luad096

**Published:** 2023-08-16

**Authors:** Sabrina Berti, Elena Luppi, Marco Seri, Guido Zavatta

**Affiliations:** Division of Endocrinology and Diabetes Prevention and Care, IRCCS Azienda Ospedaliero-Universitaria di Bologna, Italy; Department of Medical and Surgical Sciences (DIMEC), Alma Mater Studiorum University of Bologna, Bologna, Italy; Department of Medical and Surgical Sciences (DIMEC), Alma Mater Studiorum University of Bologna, Bologna, Italy; Medical Genetics Unit, IRCCS Azienda Ospedaliero-Universitaria di Bologna, Italy; Department of Medical and Surgical Sciences (DIMEC), Alma Mater Studiorum University of Bologna, Bologna, Italy; Medical Genetics Unit, IRCCS Azienda Ospedaliero-Universitaria di Bologna, Italy; Division of Endocrinology and Diabetes Prevention and Care, IRCCS Azienda Ospedaliero-Universitaria di Bologna, Italy; Department of Medical and Surgical Sciences (DIMEC), Alma Mater Studiorum University of Bologna, Bologna, Italy

**Keywords:** osteogenesis imperfecta, COL1A1 mutation, young woman, pregnancy, bone, fractures

## Abstract

Osteogenesis imperfecta (OI) is a rare heritable skeletal dysplasia, clinically characterized by abnormal bone fragility and predisposition to fractures. Here, we describe the case of a 30-year-old woman harboring a novel frameshift variant in the *COL1A1* gene, causing a mild but characteristic phenotype of type I OI. She has blue sclerae, a medical history of fractures during infancy and puberty, a vertebral fracture at a young age, and joint hypermobility. The mutation, c.108del (p.Pro37GInfs*37), causes a premature stop codon insertion, predicted to lead to an unstable mRNA, with a consequent reduction in type I collagen quantity. At present, little is known about the evolution of this phenotype during pregnancy, lactation, and premenopause, conditions that could increase the risk of fractures. Management of type I OI in a young woman of childbearing potential is problematic because most antiosteoporotic drugs are contraindicated in pregnancy, as discussed in our brief review.

## Introduction

Osteogenesis imperfecta (OI) is a group of connective tissue diseases characterized by bone brittleness. It has an estimated incidence of approximately 1 in 10 000 to 20 000 live births [1]. OI was historically classified into 4 main clinical types based on phenotype and severity. Type I is the most common and the mildest form of OI, characterized by a greater predisposition to bone fractures in otherwise asymptomatic individuals. Blue sclerae are typical, stature can be normal, but often lower than the parental target, sometimes associated with dentinogenesis imperfecta, joint hypermobility, muscle weakness, and hearing loss in adult age. Fractures, which generally heal without bone deformities, tend to decrease after puberty, whereas they can increase again in adulthood and after menopause in women. Respiratory complications are the leading cause of mortality in the most severe forms of OI, but they also represent a significant source of morbidity even in milder phenotypes because of lung architecture alteration and thoracic dysplasia. Other complications include valvular insufficiencies and aortic root dilation [1].

Currently, there are at least 20 genes related to the pathogenesis of OI. About 85% to 90% of OI cases are due to mutations in either 1 of the 2 genes encoding type I collagen chains (*COL1A1* and *COL1A2*, located at 17q21.33 and 7q21.3, respectively) and are transmitted in an autosomal dominant pattern. However, in 10% to 15% of OI cases mutations in genes encoding proteins that affect collagen folding, posttranslation modification and processing, bone mineralization, and osteoblast differentiation can be found [2].

Here, we describe the clinical and genetic features of a young woman affected by OI type I, focusing on the difficult approach to clinical management in the childbearing age.

## Case Presentation

The 30-year-old Caucasian woman was born at term with a cesarean section after a normal pregnancy, with a weight of 3600 g. She had a tibial and fibular fracture after a trauma of undetermined intensity at the age of 7 years and a fracture of her left metatarsal bone caused by minimal trauma (ankle sprain) at the age of 13 years. She also reported easy bruising and tinnitus. She had no family history of osteoporosis, and her parents are healthy; she had never smoked and denied alcohol abuse. She also reported low physical activity. Because of her fracture history, when aged 18 years, her primary care physician advised her to have a dual-energy X-ray absorptiometry, which was reported to be consistent with low bone density for her age, after which calcium and vitamin D supplements were initiated. At aged 28 years, she was referred to the Endocrine Unit of IRCCS Azienda Ospedaliero-Universitaria of Bologna, Italy, for further assessment. The following year, because of persistent low back pain, she had a magnetic resonance imaging scan of her lumbar spine, which showed a nonrecent fracture of the L2 vertebral body.

## Diagnostic Assessment

**Figure 1.**
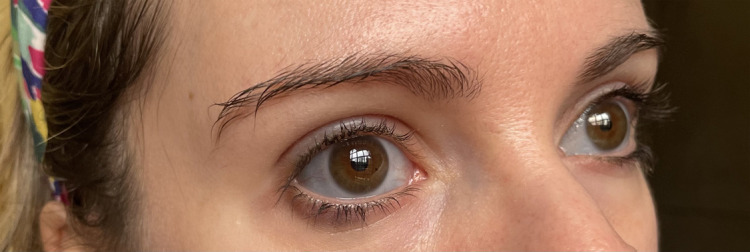
Patient's sclerae.

**Figure 2.**
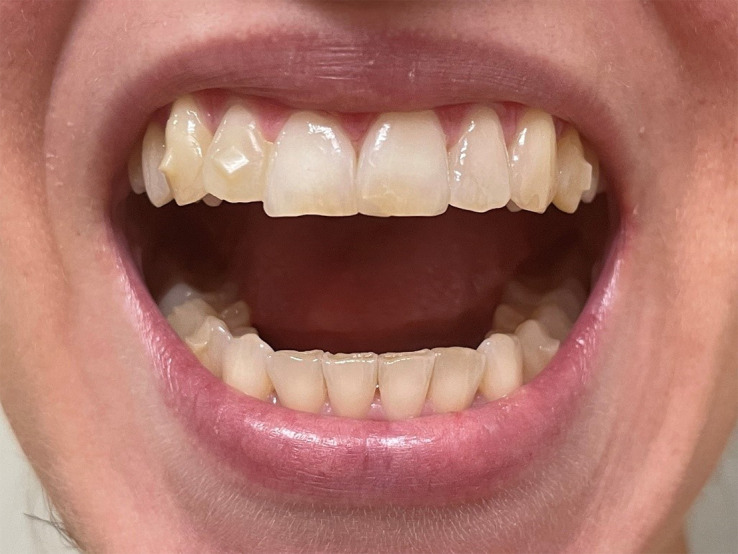
Patient's very mild alteration of dental enamel. The patient is using a clear aligner for orthodontic treatment in the superior dental arch; attachments are visible on lateral incisors, canines, and premolars.

On physical examination, she is of normal height (164.5 cm; 50th-75th percentile) and within her parental target (168 ± 8 cm); her weight is 52 kg with a body mass index of 18.6 kg/m^2^. She has blue sclerae (Fig. 1), mild alterations of tooth enamel in the lower arch (Fig. 2), hyperelastic skin, hypermobility of small joints with positive thumb and wrist signs, hyperextensibility of her elbow joint, and a mild dorsal scoliosis.

She has been on birth control continuously (ethinylestradiol 0.03 mg/chlormadinone acetate 2 mg) since the age of 18 years for contraceptive purposes and vitamin D3 880 IU + calcium carbonate 1000-mg supplements, 1 tablet every other day for at least 10 years. Low-normal serum C-terminal telopeptides of type 1 collagen were noted (Table 1). Osteocalcin, PTH, and minerals were normal.

**Table 1.** Laboratory parameters of the patient

	Normal range	02/28/2022	11/29/2021	07/28/2021	08/25/2020
PTH, pmol/L	1.3-9.3	2.4	1.8		1.8
pg/mL	(12-88)	(23)	(17)		(17)
Calcium, mmol/L	2.2-2.6	2.4	2.5	2.4	2.5
mg/dL	(8.6-10.5)	(9.4)	(9.8)	(9.6)	(9.8)
Phosphorus, mmol/L	0.8-1.5	1.3	1.2		1.2
mg/dL	(2.5-4.5)	(4.0)	(3.6)		(3.7)
Magnesium, mmol/L	0.7-1.1	0.74			
mg/dL	(1.6-2.6)	(1.8)			
Vitamin D25OH, nmol/L	50-250	65	85	80	120
ng/mL	(20-100)	(26)	(34)	(32)	(48)
C-terminal telopeptide of type 1 collagen (CTX), ng/mL	0.112-0.738	0.075	0.061		0.163
Bone alkaline phosphatase (BAP), µ/L	4.7-27	13.9	16.9		12.4
Osteocalcin, ng/mL	10-46	21			
Albumin, µmol/L	507-725	606	613	611	626
g/L	(35-50)	(41.8)	(42.3)	(42.2)	(43.2)
Urinary calcium, mmol/24 hours	1.25-10	3.8	3.5		2.6
mg/24 hours	(50-400)	(153)	(140)		(104)
Urinary phosphorus, g/24 hours	0.4-1.3	0.5	0.4		0.5
Urinary creatinine 24 hours, g/24 hours	8.8-13.3	8	6.2		8.8
g/24 hours	(1.0-1.5)	(0.9)	(0.7)		(1.0)

Laboratory parameters are expressed both in SI units and in conventional units.

Abbreviations: BAP, bone-specific alkaline phosphatase; CTX, C-terminal telopeptide of type-1 collagen.

**Figure 3.**
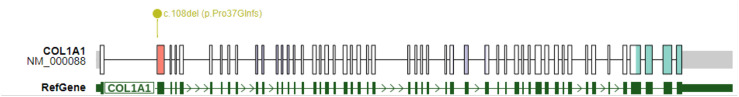
*COL1A1* gene displaying the frameshift variant identified in the patient. Each box corresponds to 1 of 51 exons of *COL1A1*. The figure was created using ProteinPaint (https://proteinpaint.stjude.org). Matching protein domains: von Willebrand factor type C domain (red), collagen triple helix repeat (violet), fibrillar collagen C-terminal domain (blue).

Because of her clinical features, a genetic consultation was performed. With informed consent, a blood sample was collected for molecular analysis of *COL1A1* gene as a first-tier test. All coding exons, including the exon-intron boundaries, were screened as described by Maioli et al [3] and DNA variants were reported according to the Gene Bank Reference Sequence NM_000088.4. This analysis detected the heterozygous frameshift variant c.108del (p.Pro37Glnfs*37) of *COL1A1*, resulting in a premature stop codon insertion predicted to cause mRNA non-sense-mediated decay (Fig. 3). This leads to a quantitative defect of collagen type 1. *COL1A1* gene shows substantial allele heterogeneity as 1065 unique variants are recorded in LOVD (https://databases.lovd.nl/shared/genes/COL1A1) and more than 1500 in Clinvar (https://www.ncbi.nlm.nih.gov/clinvar/). To our knowledge, the c.108del variant has not previously been described nor is it contained in OI databases and it is absent in the gnomAD (https://gnomad.broadinstitute.org) healthy population database. Furthermore, quantitative defects, such as frameshift variants, usually tend to result in a milder phenotype [3] consistent with our patient's clinical features. Therefore, the c.108del variant in *COL1A1* can be considered OI causative.

As a result of this genetic diagnosis a multidisciplinary follow-up was carried out.

**Figure 4.**
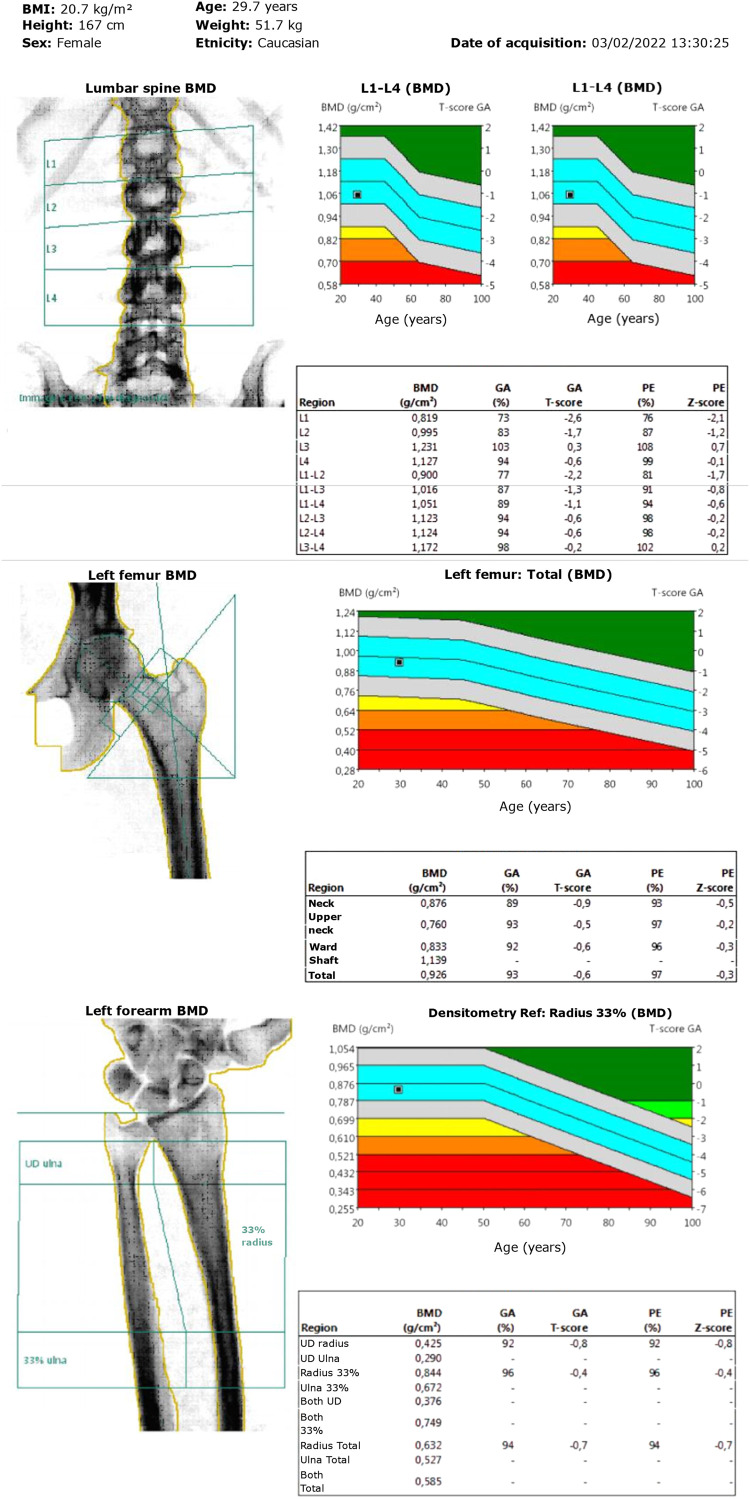
Lumbar, femoral, and distal forearm 2022 dual-energy X-ray absorptiometry, showing normal bone mineral density at all sites.

**Figure 5.**
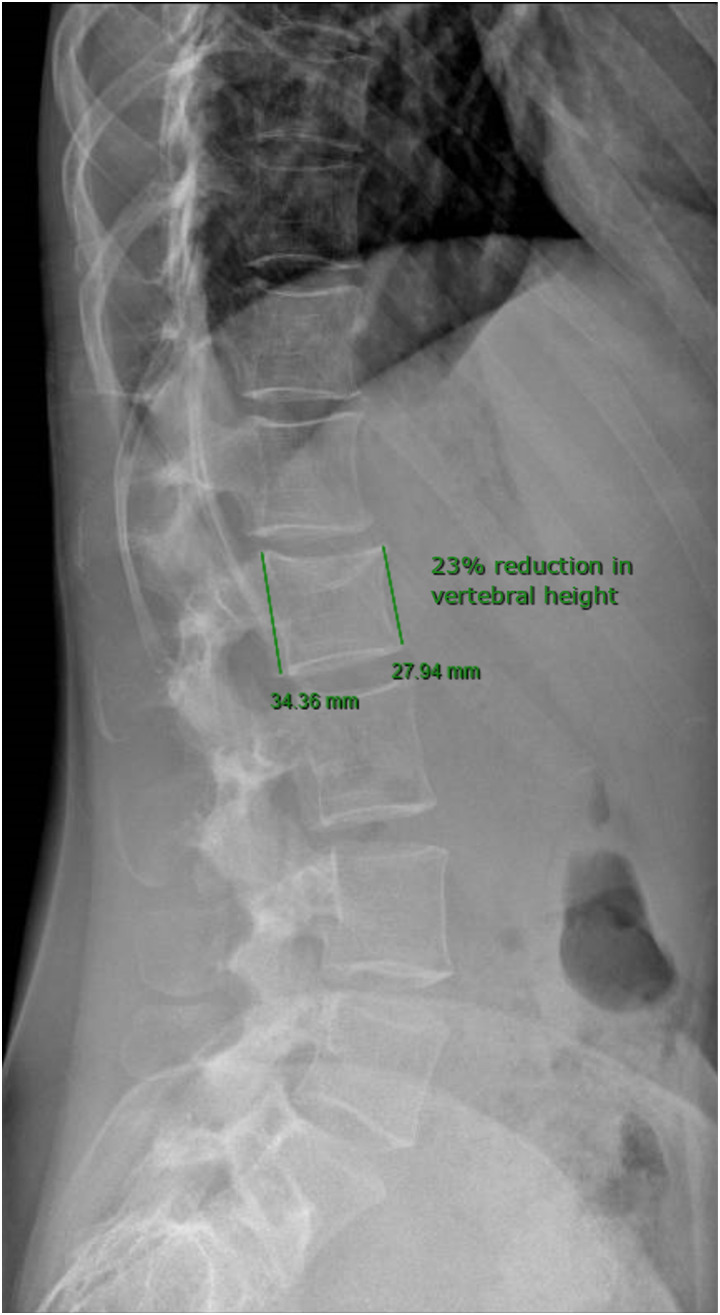
Thoracic and lumbar spine lateral view X-ray showing a mild fracture of L2 vertebral body without other vertebral deformities.

The patient had lumbar dual-energy X-ray absorptiometry scans in 2020 and 2022, showing bone mineral density (BMD) within the expected range for age (Fig. 4); L2 was excluded from the analysis because it was fractured. X-rays of the thoracic and lumbar spine for vertebral morphometry confirmed one mild vertebral fracture of L2 and no other vertebral deformities (Fig. 5). Her daily dietary calcium intake, assessed using the International Osteoporosis Foundation Questionnaire *Calcium Calculator*, was estimated to be 780 mg/day.

Audiometry, tympanometry, and auditory brainstem response were normal. A transthoracic echocardiogram revealed the redundancy of mitral leaflets with a tendency to prolapse and minimal-mild mitral insufficiency, along with redundant interatrial septum without shunting. She regularly has dental check-ups, which have not revealed any caries.

Because of the autosomal dominant mode of inheritance of OI (50% chance of passing on the altered gene copy to future children), a preconception genetic counseling was performed, discussing preimplantation genetic diagnosis, prenatal invasive, and noninvasive monitoring techniques.

## Treatment

Because of her mild clinical features, the patient's desire for a pregnancy and considering the suboptimal dietary daily calcium intake, we suggested continuing the treatment with calcium and vitamin D supplements alone.

## Outcome and Follow-Up

Thirteen months since the genetic diagnosis, no adverse events or complications (including new fractures) have occurred. The next follow-up appointment will be scheduled 18 months after the last visit or when she gets pregnant.

## Discussion

In our patient, a novel *COL1A1* mutation was identified, thus broadening the genotypic spectrum of OI. The genotype was consistent with her phenotype, encompassing mildly reduced BMD, only 1 asymptomatic mild vertebral fracture, previous tibial, fibular, and metatarsal fractures.

The detection of a morphometric vertebral fracture (L2) could suggest that the approach with only calcium and vitamin D3 supplements recommended by her general practitioner may have been too conservative or potentially ineffective. In any case, the patient's desire for pregnancy should prompt the clinician to carefully weigh the benefits and risks of more aggressive pharmacological approaches.

There are currently no medical treatments for OI approved by the US Food and Drug Administration (FDA) or the European Medicines Agency. Neridronate is the only authorized medication for OI in Italy. OI is usually managed by sequential treatments of oral or IV bisphosphonates, calcium and vitamin D supplements, physical aids and physical therapy, orthotics, or corrective surgery. Screening for pulmonary complications (with spirometry and single-breath CO-transfer with helium dilution at least once every 3 years) as well as screening of cardiac complications and regular hearing and dental check-ups are recommended. Combined oral contraceptives can be used too, when needed, because they have been associated with a significant decrease in fracture risk in premenopausal women [4].

We cannot predict the disease evolution in our patient during pregnancy, lactation, and premenopause, which are conditions that might increase fracture risk [4]; however, recent data from the Danish Health registers on fractures following pregnancy in OI patients seem reassuring [5].

Management of women of childbearing age affected by type I OI still remains a gray area, and careful management in drug prescriptions is required to limit teratogenic effects. To gain insights into the available evidence, we carried out a narrative review of the current pharmacological treatment options.

Patients with OI are commonly treated with bisphosphonates (usually pamidronate, alendronate, neridronate). These are the mainstays of pharmacologic treatment in pediatric patients with OI because they can improve bone mass and architecture in growing children [1]. However, according to the most recent Cochrane reviews, in adults with OI, the beneficial effects of bisphosphonates on clinical status and fracture rates are not conclusive [6].

Moreover, the use of bisphosphonates is contraindicated in pregnancy (FDA Pregnancy Category C—risk cannot be ruled out). Although some new data suggest that bisphosphonates might be safe in women with childbearing potential [7], caution is still needed [4].

Denosumab, a human monoclonal anti-RANKL antibody, has been investigated in children with severe forms of OI with conflicting results, mainly because of the significant risks of hypercalcemia, hypercalciuria, and rapid bone loss following treatment discontinuation [8].

Overall, considering the lack of consistent evidence regarding the safety and efficacy of denosumab in type I OI, this option did not seem a suitable choice for this patient.

Teriparatide, the recombinant (1-34) N-terminal active peptide fragment of PTH, was found to be superior to bisphosphonates in the prevention of vertebral fractures in postmenopausal osteoporosis. In the setting of OI, the evidence is more limited. Orwoll et al showed teriparatide significantly increased BMD and improved biochemical markers in adults with OI type I compared with placebo. However, in the 18-month period of observation, the difference in fracture rate between the 2 groups was not significant [9]. Teriparatide was more effective than neridronate in adults with type I OI in terms of BMD gain in the lumbar spine and regarding quality of life. New fragility fractures occurred in fewer patients in the teriparatide group than in the neridronate group during a 24-month period, but this difference was not significant [10].

No safety data for teriparatide during pregnancy are available (FDA Category C). However, unlike bisphosphonates, the short half-life of the drug should not theoretically carry major risks for the fetus. Teriparatide use in the case of young women is off-label and in some countries potentially not covered by insurance. Moreover, on discontinuation, antiresorptive agents are recommended to maintain BMD. There are no data available on abaloparatide use in OI.

In conclusion, teriparatide improves BMD in type I OI but does not significantly reduce the risk of fractures. Nevertheless, given the rarity of OI and the limitations in measuring fracture endpoints in this condition, it would be our preferred treatment option in case of recent or recurrent major clinical fractures.

Anti-sclerostin antibodies may hold some promise for improving BMD without increasing brittleness in patients with OI [2], and setrusumab has successfully overcome phase 2 trials in humans. Another study (NCT04545554) is ongoing to assess the pharmacokinetics, safety, and efficacy of romosozumab in young patients with OI. However, because of the lack of conclusive clinical evidence, antisclerostin agents are not yet a viable option for our patient.

Promising data in murine models are emerging on anti-TGF-β antibodies (fresolimumab), and a safety trial is currently ongoing (NCT03064074).

In conclusion, in our 30-year old female patient harboring a novel *COL1A1* mutation, aside from calcium and vitamin D supplements, other medical options (bisphosphonates, denosumab, and teriparatide) are of limited use and would be contraindicated in pregnancy. This case is challenging because the presence of a woman of childbearing potential with OI may lead the clinician not to prescribe a treatment that could be beneficial in managing the disease but that may also be teratogenic in case of pregnancy. By contrast, male counterparts of the same age as our patient might be more likely to receive early antiosteoporotic treatments.

## Learning Points

- Osteogenesis imperfecta (OI) should be suspected in cases of fractures caused by minimal-mild trauma in young subjects, especially when associated with low bone mineral density (BMD) and blue sclerae.
- OI has a broad phenotypic and genotypic spectrum: mutations of *COL1A1* and *COL1A2* are the most common and new mutations are constantly being discovered.
- Currently, treatment options for young women with type I OI are scarce, with teriparatide seeming to be the safest and most effective therapy to increase BMD, although no significant fracture risk reduction was demonstrated. Clinical severity of the disease should guide the decision to start the drug, especially in case of recurrent or recent fractures. The optimization of calcium and vitamin D3 intake through diet or supplements is a less effective alternative. Prompt discontinuation of teriparatide is recommended in case of pregnancy.
- Bisphosphonates and denosumab are other medical options; efficacy and safety of antisclerostin agents and anti-TGF-β antibodies are still being evaluated in clinical trials.

## Data Availability

Original data generated and analyzed for this case report are included in this published article. The result of genetic analysis has been submitted to Clinvar database with accession number SCV003929406.

## Abbreviations

BMD: bone mineral density
FDA: Food and Drug Administration
OI: osteogenesis imperfecta
